# Supplementary material for: The Mediating Effect of Cytokines on the Association between Fungal Sensitization and Poor Clinical Outcome in Asthma
Source: Biomedicines. 2022 Jun 19;10(6):1452. doi: 10.3390/biomedicines10061452 (PMC9220002; doi:10.3390/biomedicines10061452)
Supplement: Supplementary file 1 [file biomedicines-10-01452-s001.zip › biomedicines-1778028-supplementary.pdf]

Supplemental Table S1

	**Male (N=39)**	**Female (N=58)**	**P-values**
**IL-17A level**	0.328±0.422	0.417±0.548	0.369
**Correlation between IL-17A and Candida albicans**	0.233	0.458	0.166
